# Aerobic Training Prevents Heatstrokes in Calsequestrin-1 Knockout Mice by Reducing Oxidative Stress

**DOI:** 10.1155/2018/4652480

**Published:** 2018-04-03

**Authors:** Flávia Alessandra Guarnier, Antonio Michelucci, Matteo Serano, Laura Pietrangelo, Claudia Pecorai, Simona Boncompagni, Feliciano Protasi

**Affiliations:** ^1^Center for Research on Ageing and Translational Medicine (CeSI-MeT), University G. d'Annunzio, 66100 Chieti, Italy; ^2^Department of Neuroscience, Imaging, and Clinical Sciences (DNICS), University G. d'Annunzio, 66100 Chieti, Italy; ^3^Department of General Pathology, Londrina State University, 86057-970 Londrina, PR, Brazil; ^4^Department of Medicine and Aging Science (DMSI), University G. d'Annunzio, 66100 Chieti, Italy

## Abstract

Calsequestrin-1 knockout (CASQ1-null) mice suffer lethal episodes when exposed to strenuous exercise and environmental heat, crises known as exertional/environmental heatstroke (EHS). We previously demonstrated that administration of exogenous antioxidants (*N*-acetylcysteine and trolox) reduces CASQ1-null mortality during exposure to heat. As aerobic training is known to boost endogenous antioxidant protection, we subjected CASQ1-null mice to treadmill running for 2 months at 60% of their maximal speed for 1 h, 5 times/week. When exposed to heat stress protocol (41°C/1 h), the mortality rate of CASQ1-null mice was significantly reduced compared to untrained animals (86% versus 16%). Protection from heatstrokes was accompanied by a reduced increase in core temperature during the stress protocol and by an increased threshold of response to caffeine of isolated *extensor digitorum longus* muscles during *in vitro* contracture test. At cellular and molecular levels, aerobic training (i) improved mitochondrial function while reducing their damage and (ii) lowered calpain activity and lipid peroxidation in membranes isolated from sarcoplasmic reticulum and mitochondria. Based on this evidence, we hypothesize that the protective effect of aerobic training is essentially mediated by a reduction in oxidative stress during exposure of CASQ1-null mice to adverse environmental conditions.

## 1. Introduction

Hyperthermia is an abnormal rise in body temperature above the hypothalamic set point caused by excessive accumulation of external (environmental) or internal (metabolic) heat. When the core body temperature rises above 40°C, hyperthermia may result in heatstroke, a life-threatening episode characterized by dysfunction of central nervous system and peripheral organs [1].

Malignant hyperthermia (MH), identified and described for the first time in 1960 [2], is an inherited pharmacogenetic disorder that manifests as a life-threatening hypermetabolic response to the administration of volatile anesthetics such as halothane or isofluorane [2, 3]. The main clinical features of MH crises include uncontrolled muscle contracture, rupture of muscle fibers (i.e., rhabdomyolysis), increased circulating levels of creatine kinase (CK) and K^+^, and increased oxygen consumption [3]. Interestingly, hyperthermic crises known as exertional/environmental heatstroke (EHS), but virtually identical to anesthetic-induced MH episodes, have also been reported in humans exposed to elevated environmental temperatures or strenuous exercise performed in challenging conditions [1, 4–6].

Most of families (70–80%) affected by MH susceptibility present mutations in the *RYR1* gene [7], which encodes for a 2200 kDa protein forming the sarcoplasmic reticulum (SR) Ca^2+^-release channel of skeletal muscle, the ryanodine receptor type-1 (RyR1) [8, 9]. An association between *RYR1* variants and exertional- or heat-induced rhabdomyolysis and sudden death has been reported [10–13]. The correlation between MH and EHS is also supported by evidence collected in animal models: (a) in *porcine stress syndrome* (PSS), swine carrying a point mutation in *RYR1* trigger MH episodes in response to halothane administration but also following exposure to either heat or emotional/physical stress [14, 15]; (b) knock-in mice carrying gain-of-function point mutations in *RYR1* linked to MH in humans exhibit heat- and anesthetic-induced MH episodes [16, 17]. In addition, we discovered that male mice lacking calsequestrin-1 (CASQ1-null), the main Ca^2+^-binding protein located in the lumen of SR terminal cisternae that modulates RyR1 opening probability [18–20], exhibit lethal hyperthermic episodes when exposed to anesthetics, heat, and strenuous exercise [18, 21–23].

The molecular mechanisms underlying rhabdomyolysis of skeletal muscle fibers during MH/EHS crises appear to be complex cascade of events revolving around an increased leak of Ca^2+^ from the mutated RyR1 [24–28] and an excessive production of reactive oxygen/nitrogen species (ROS/RNS) [24]. Michelucci et al. [23] demonstrated that administration of antioxidants (*N*-acetylcysteine and trolox) protects CASQ1-null mice from anesthetic- and heat-induced lethal crises by reducing mitochondrial production of superoxide anion and global oxidative stress.

Oxidative stress levels in a cell are the net result of production and removal of oxidative species since the early 80s, it has been demonstrated that aerobic training promotes mitochondrial biogenesis in skeletal muscle while boosting endogenous antioxidant levels [29]. It seems that, although free radical production increases during exercise [30], their rise may act as a signal leading to both upregulation and increased activity of antioxidant enzymes [31–33].

In the present study, we hypothesized that aerobic training, by boosting antioxidant defenses [31–33], could reduce mortality of CASQ1-null mice during hyperthermic crisis. The results collected in this study indicate that training effectively protects CASQ1-null mice from EHS, an effect essentially mediated by a significant reduction in oxidative stress.

## 2. Materials and Methods

### 2.1. CASQ1-Null Male Mice

CASQ1-null mice were generated as previously described [27]. All animals used in this study were males, as this gender is more susceptible to MH/EHS-like crises when exposed to halothane and heat [22]. Mice were housed in microisolator cages at 20°C in a 12 h light/dark cycle, provided free access to water and food. Age-matched C57BL/6 (WT) mice were used as controls. All experiments were conducted according to the Directive of the European Union 2010/63/UE and were approved by the Italian Ministry of Health (1199/2015-PR).

### 2.2. *In Vivo* Experiments

#### 2.2.1. Incremental Test

This protocol consisted of a single session of exercise on a treadmill with no incline, according to the protocol described by Cunha et al. [34] and Gladden and Hogan [35], performed at room temperature of 20 ± 2°C. A mild electrical stimulus (0.5 mA) was applied to mice that stepped off the treadmill to keep them exercising. The test started with a warm-up of 10 min at a speed of 6 m/min. The speed of the treadmill was then increased by 3 m/min (from 6 to 39 m/min) every 3 min until exhaustion, defined as the time when the mice were no longer able to maintain regular gait. Workloads corresponding to 85 and 60% of peak workload were determined for each mouse.

#### 2.2.2. Constant Load Test and Lactate Measurements

This protocol consisted of a warm-up period of 10 min at 6 m/min followed by a 28 min constant load running on a treadmill with no incline (at 85% of maximal speed reached in the incremental test) [33], performed at room temperature of 20 ± 2°C. Blood samples (~50 *μ*L) were collected from the tail vein while mice were kept running, every 7 min. Blood was then transferred to 1.5 mL microtubes, centrifuged at 3000 ×g for 15 min at 4°C for plasma separation, and stored in 200 *μ*L microtubes at −20°C. The lactate concentration in the blood was analyzed in plasma using a colorimetric enzymatic assay kit (Lactate Assay Kit II; Sigma-Aldrich®, St. Louis, MO, USA), following the manufacturer's instructions. The lactate concentration in the colorimetric reaction was measured spectrophotometrically at 450 nm (Spectra MAX 190; Molecular Devices, Sunnyvale, CA, USA) and expressed as mmol of lactate/L.

#### 2.2.3. Grip Strength Test

The strength developed by mice during instinctive grasp was measured as previously described [23, 36, 37]. Briefly, mice were held by the tail and allowed to grasp a metal grating connected to a Shimpo Fgy 0.5X transducer (Metrotec Group, Spain). Once the mouse had firmly grasped the grating, a steady and gentle pull was exerted on the tail. Measurements of the peak force generated by each mouse using forelimbs were repeated three times with appropriate intervals (about 30 s) to avoid fatigue. Average peak force values were then normalized to the total body mass.

#### 2.2.4. Aerobic Training Protocol

**Figure 1:**
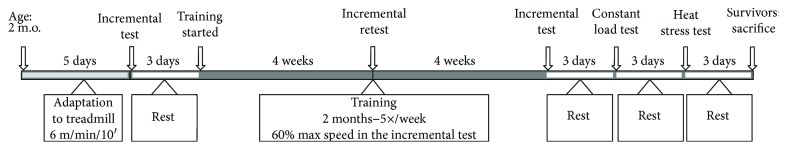
Schematic view of the experimental protocol. Two months old CASQ1-null male mice were randomly selected to perform 2 months of aerobic training. Control WT and untrained (control) CASQ1-null mice were always subjected to identical procedures, without the aerobic training. m.o.: months old.

WT (*n* = 7) and CASQ1-null (*n* = 21) mice were enrolled in the study at 2 months of age after weight and grip strength were measured. Each mouse was then (a) accustomed for 5 days to treadmill running (Columbus Instruments, Columbus, OH, USA), (b) subjected to the incremental test (see above), and (c) randomly assigned to the CASQ1-null training (*n* = 7) or to the control groups (*n* = 14). The training group of CASQ1-null mice was subjected to aerobic training [33, 34] on the treadmill at 60% of their individual maximal speed reached during the incremental test. After 4 weeks, trained CASQ1-null mice performed a new incremental test to readjust the training load and guarantee the 60% of workload in the following 4 weeks. At the end of 8 weeks of training, body weight and grip strength were reassessed for all mice (WT and CASQ1-null control and trained), which were also reevaluated with the incremental test. After 2 days of rest, all animals additionally performed a constant load test (see above) with blood collection, to measure the lactate accumulation/removal ratio. A detailed overview of experimental protocol is shown in Figure 1.

#### 2.2.5. Heat Stress Protocol and Core Temperature Recording

Three days after the constant load test (see above), all mice were subjected to a heat stress protocol. Animals were placed in an environmental chamber in which temperature was maintained at 41°C, 1 h [21]. During exposure to heat, core body temperature was measured using a rectal thermometer taped to the tail of the animals and recorded every 5 min throughout the duration of heat challenge. Breathing and spasmodic contractions were visually monitored, while muscle rigidity was manually confirmed by limb resistance immediately after animal death. Surviving animals were returned to normal housing conditions and monitored for 24 h to assess possible delayed deaths. After 3 days, the mice that survived from the heat stress protocol were sacrificed, and muscle samples collected and processed for further analysis.

### 2.3. Ex Vivo and *In Vitro* Experiments

#### 2.3.1. *In Vitro* Contracture Test (IVCT) in (EDL) Muscles

Intact EDL muscles were dissected from hind limbs of mice, placed in a dish containing Krebs-Henseleit solution, and pinned and tied with fine silk sutures at each end. Muscles were then mounted vertically between two platinum electrodes immersed in an organ chamber filled with Krebs-Henseleit solution and attached to a servomotor and force transducer (model 1200A; Aurora Scientific, Aurora, ON, Canada). Before starting the experimental protocol, stimulation level and optimal muscle length (*L*_0_) were determined using a series of 80 Hz train stimulus in order to adjust the muscle to the length that generated maximal force (*F*_0_). During the experiments, temperature was kept constant at 25°C. To determine caffeine sensitivity of resting tension, EDL muscles were subjected to an *in vitro* contracture test (IVCT) as previously described [23]. Briefly, isolated EDL muscles were continuously stimulated at 0.2 Hz at 23–25°C, caffeine concentration in the bath was changed every 3 minutes (no wash between applications) as follows: 2, 4, 6, 8, 10, 14, 18, and 22 mM. Muscle basal tension was measured at the end of each step of caffeine application and reported both as specific and relative force. Specific force was calculated by normalizing the absolute force to the cross-sectional area of the muscle.

#### 2.3.2. Preparation of Total Homogenates and Isolation of Mitochondria and SR Membranes

Total homogenates were prepared by placing muscles *tibialis anterior* (TA) and *gastrocnemius* on ice in an Ultra-Turrax homogenizer (2 × 30 s at 14,500 rpm) containing 50 mg/mL of tissue in 10 mM KH_2_PO_4_/K_2_HPO_4_ buffer and 120 mM KCl at pH 7.4 [38]. Total homogenate preparations were used for assessment of total diene conjugates production, protein carbonylation, and calpain activity assays (see below).

Mitochondria and SR membranes were isolated from gastrocnemius to assay-specific organelle lipid peroxidation. Samples were placed in an Ultra-Turrax homogenizer (2 × 30 s at 14,500 rpm) in 5 vol of 30 mM KH_2_PO_4_, 5 mM EDTA, 3.0 M sucrose, 0.5 mM dithiothreitol, 0.3 mM phenylsulfonyl fluoride, and 1% (v/v), 1 *μ*M leupeptin, 1 *μ*M pepstatin (pH 6.8). All steps for isolation were performed at 4°C. Mitochondrial fraction was prepared from the total homogenates by differential centrifugation as previously described [39]. The supernatant from a first homogenate centrifugation (1000 ×g for 10 min) was centrifuged at 14,000 ×g for 35 min. The pellet was then suspended in 30 mM imidazole, 60 mM KCl, and 2 mM MgCl_2_ (pH 7.0) and stored at −80°C until use. This resuspension was then used to assay cytochrome *c* oxidase activity and lipid peroxidation in mitochondrial membranes. Microsomes vesicles were prepared by isoelectric precipitation from the supernatant of mitochondrial isolation as previously described [40, 41]. Sodium acetate (1 mM) was added to the samples until the pH was 4.0 and then centrifuged at 10,000 ×g for 10 min. The supernatants were discharged, and pellets were resuspended in the same volume that was initially used, of KCl 1.15% glycerol (4 : 1 v/v). The pellets were mechanically broken using a vortex and then centrifuged again in 10,000 ×g for 10 min. The final pellet was resuspended in 100 mM KH_2_PO_4_ and glycerol (4 : 1 v/v), mixed using a vortex, and stored in microtubes at −80°C. For Ca^2+^-dependent ATPase activity, the pellets were resuspended in 3% polyethylene glycol, 5 mM azide, 80 mM KCl, and 0.1 mM ouabain (pH 7.5). Total proteins were quantified by the method of Bradford [42], using bovine serum as a standard.

#### 2.3.3. Determination of Ca^2+^ ATPase Activity

Activity of sarco/endoplasmic Ca^2+^ ATPase (SERCA), the main SR Ca^2+^ pump of skeletal muscle, was estimated in SR isolated membranes from gastrocnemius by using a colorimetric assay that quantifies the amount of inorganic phosphate (P_i_) that complexes with ammonium molybdate and malachite green following release from SERCA-mediated ATP hydrolysis [43, 44]. Briefly, the reagent to quantify P_i_ was prepared by mixing 1 vol of 10% (w/v) ammonium molybdate in 4 M HCl with 3 vol 0.2% (w/v) malachite green in 4 M HCl. The reaction medium, composed by 2 mM EDTA, 10 mM CaCl_2_, 2 mM MgCl_2_, and 2 mM ATP, was mixed to malachite green/ammonium molybdate dye reagent, and then the reaction was started with the addition of 300 *μ*g protein/mL of membranes resuspension. When P_i_ was complexed with ammonium molybdate and malachite green in 4 M HCl, it creates a green color which was quantified by reading the absorbance spectrophotometrically at 660 nm and compared to a standard curve of known P_i_ concentrations (0–15 nmols of PO_4_^−2^ from NaPO_4_). The color formation was monitored for 5 min, the P_i_ concentration at every minute was calculated using a standard curve, and the differences are considered the activity of Ca^2+^ ATPase pump [44]. All the reactions were repeated in the presence of thapsigargin (100 nM), an inhibitor of the SERCA family of Ca^2+^ pumps [45], to differentiate SERCA activity from any other ATP-dependent activity that could interfere with the measurement.

#### 2.3.4. Determination of Calpain Activity

The activity of calpain [46, 47] was measured in total homogenates from gastrocnemius muscle, by a chemiluminescence assay using a calpain protease assay kit (Calpain-Glo Protease Assay®; Promega, Madison, WI, USA). The assay provides a proluminescent calpain substrate, in a buffer system optimized for calpain and luciferase activities. During the assay, calpain cleavage of the substrate generates a glow-type luminescent signal produced by the luciferase reaction. In this homogeneous, coupled-enzyme format, the signal is proportional to the amount of calpain activity present in the sample [48]. Muscle homogenates were prepared as described above, diluted to a concentration of 6.25 mg/mL in 10 mM KH_2_PO_4_ buffer, pH 7.4 in 0.9% NaCl, and finally processed according to the manufacturer's instructions. Results were expressed as calpain activity/mg of muscle tissue.

#### 2.3.5. Preparation of Samples for Electron Microscopy (EM)

EDL muscles were dissected from sacrificed animals, pinned on a Sylgard dish, fixed at room temperature with 3.5% glutaraldehyde in 0.1 M sodium cacodylate (NaCaCo) buffer (pH 7.2), and then stored in the fixative at 4°C. Small bundles of fixed tissue were then postfixed, embedded, stained en bloc, and sectioned for EM as described previously [49]. Ultrathin sections (~50 nm) were then cut in a Leica Ultracut R microtome (Leica Microsystem, Austria) using a Diatome diamond knife (Diatome Ltd. CH-2501 Biel, Switzerland). Sections were examined at 60 kV (after double staining with uranyl acetate and lead citrate) with a FP 505 Morgagni Series 268D electron microscope (FEI Company, Brno, Czech Republic), equipped with a Megaview III digital camera (Munster, Germany) and Soft Imaging System (Munster, Germany).

#### 2.3.6. Quantitative EM Analysis of Mitochondrial Volume and Damage

(A) Mitochondrial volume was determined using the well-established stereology point-counting technique [50, 51] in EM micrographs collected from transverse sections of samples at 8900× magnification. Briefly, after superimposing an orthogonal array of dots at a spacing of 0.20 *μ*m to the electron micrographs, the ratio between numbers of dots falling within mitochondrial profiles and total number of dots covering the whole image was used to calculate the relative fiber volume occupied by mitochondria. (B) In the same set of micrographs, the number of severely damaged mitochondria was evaluated and reported as percentage of the total number. Mitochondria with one of the following ultrastructural alterations were classified as severely damaged: (a) presenting disruption of the external membrane, (b) presence of internal vacuolization and/or disrupted internal cristae, and (c) containing myelin figures.

#### 2.3.7. Cytochrome *c* Oxidase Activity

Cytochrome *c* oxidase activity was measured in isolated mitochondria from gastrocnemius muscles, by a colorimetric assay kit (Cytochrome *c* Oxidase Assay Kit; Sigma-Aldrich, St. Louis, MO, USA) based on the observation of the decrease in absorbance at 550 nm of ferrocytochrome *c* caused by its oxidation to ferricytochrome *c* by cytochrome *c* oxidase [52]. All samples used in the assay had the same amount of protein (300 *μ*g protein/mL), and the results were expressed as cytochrome *c* oxidase activity (U/mL × 10^−3^).

#### 2.3.8. Oxidative Stress Measurements

Protein carbonyl group formation is a classic and immediate biomarker of oxidative modification to proteins [53, 54]. Here, carbonyl protein content was measured with a modified version of a protocol previously described [53]. Briefly, TA (50 mg/mL) was homogenized in 50 mM of phosphate buffer, 1 mM ethylenediaminetetraacetic acid, pH 7.4. Samples were then centrifuged at 600 ×g for 10 min at 4°C. A volume of 200 *μ*L of 2,4-dinitrophenylhydrazine (DNPH) was added to 200 *μ*L of supernatant and incubated at room temperature. After 30 min of incubation, 100% trichloroacetic acid (TCA) was added and samples were placed on ice for 5 min and then spinned at maximal speed for 2 min. Supernatants were discarded, while pellets were washed in cold acetone and placed at −20°C for 5 min. Then, acetone was carefully removed, and pellets were resuspended in 0.5 mL 6 M guanidine hydrochloride to be spectrophotometrically read at 375 nm. To calculate the protein carbonyl content, the following formula was used: C = [(OD 375 nm)/6.364 × 100] nmol/well, where 6.364 is the extinction coefficient using the enclosed 96-well plates in mM (=22 mM^−1^ cm^−1^ × 0.2893 cm path length in well). Results were expressed as nmol carbonyl/mg of total protein, which were quantified in each sample at 280 nm.

Oxidation of fatty acids forms conjugated dienes that absorb UV light at 230 to 235 nm. Measurement of dienes is a useful index of peroxidation in pure lipids or isolated lipoproteins and has the advantage that it measures early stages in peroxidation [55]. Muscle homogenate and SR vesicles were dispensed in concentration of 20 *μ*g/mL protein in solution with 10 mM phosphate buffer containing 1% Lubrol [56]. The absorption spectrum was then recorded. The rate of conjugated diene formation was estimated according to the lipid oxidation index, A_233nm_/A_215nm_, which provides a sensitive method for determination of lipid peroxidation [57].

### 2.4. Statistical Analysis

The statistical analysis is reported in the legend of each figure. Mean ± SD was used when variability representative values are needed (higher number of samples/animals), while mean ± SEM was used to represent precision of a measurement (lower number of samples/animals).

## 3. Results

### 3.1. Aerobic Training Increased Functional Output and Aerobic Capacity of CASQ1-Null Mice

Before the 2 months training protocol, and two days after the last training session, body weight and grip strength were evaluated in each mouse (Table 1). Body weight between WT and CASQ1-null mice, although slightly lower in the latter at both 2 and 4 months of age, was not significantly different. Significant differences were observed for the functional output, as the grip strength test revealed that untrained CASQ1-null mice were about 60% weaker than age-matched WT at both 2 and 4 months of age. On the other hand, the maximum speed reached during the incremental test was surprisingly higher in CASQ1-null mice: this last result is likely due to the increased number and volume of mitochondria in CASQ1-null muscles (see below; see also [27]). Two months of aerobic training (2 to 4 months of age; see Figure 1), while did not change the average body weight of CASQ1-null mice, significantly improved both functional parameters: (a) the grip strength output normalized by weight increased from 3.0 ± 0.8 g/g of body weight in untrained CASQ1-null to 4.8 ± 1.1 g/g of body weight in trained CASQ1-null and (b) the maximum speed reached during the incremental test was slightly higher in trained than in untrained CASQ1-null mice, respectively, 32.5 ± 3.9 versus 28.1 ± 2.7 m/min.

**Figure 2:**
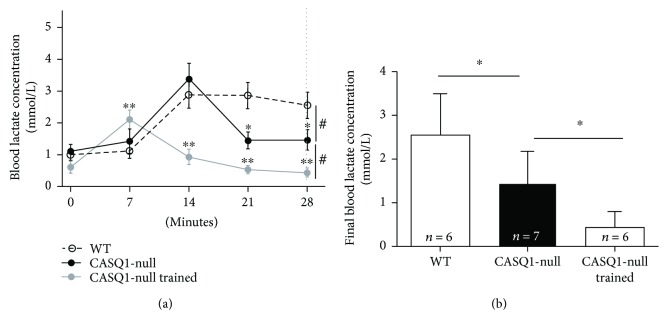
Accumulation of lactate in the bloodstream during constant load test. (a) Lactate accumulation in the blood during a 28-minute constant load test (at 85% of maximal speed reached during the incremental test). The vertical dashed line represents the time point in which final blood lactate concentration (plotted in panel (b)) was evaluated. ^#^*p* < 0.05, as evaluated by two-way ANOVA (differences among paired curves); ^∗^*p* < 0.05, CASQ1-null versus WT, and ^∗∗^*p* < 0.05, CASQ1-null versus CASQ1-null trained, as evaluated by Bonferroni's post hoc test (indicating differences among the same time point). (b) Average blood lactate concentration at the end of incremental test (28th minute; dashed line in panel (a)). Data are given as mean ± SD; ^∗^*p* < 0.05, as evaluated by one-way ANOVA followed by Tukey's post hoc test. *n* = number of mice.

As lactate accumulation/removal ratio is considered a good marker of aerobic capacity [58, 59], we subjected all mice at 4 months of age to a 28-minute constant load test (at 85% of the maximal speed reached during the incremental test) and measured the lactate accumulation in the bloodstream every 7 minutes (Figure 2(a)). WT mice displayed a typical lactate accumulation curve, increasing from baseline (0.09 ± 0.30 mmol/L) to a peak at the 14th minute of 2.87 ± 0.90 mmol/L, followed by a plateau. On the other hand, the lactate levels in the bloodstream in untrained CASQ1-null mice (1.11 ± 0.46 at baseline) reached a peak of 3.38 ± 1.10 mmol/L at the 14th minute, but then declined in the second part of the experiment. This decay was even greater in trained CASQ1-null mice, with a peak of 2.10 ± 0.73 mmol/L at 7th minute followed by a constant decline. Indeed, the blood lactate concentration at the end of the constant load test (28th minute; dashed line in Figure 2(a)) exhibited a reduction compared to WT mice of, respectively, ~39% and ~70% in untrained and trained CASQ1-null animals (Figure 2(b)).

### 3.2. Aerobic Training Decreased Mitochondrial Damage and Improved Mitochondrial Function in CASQ1-Null Mice

**Figure 3:**
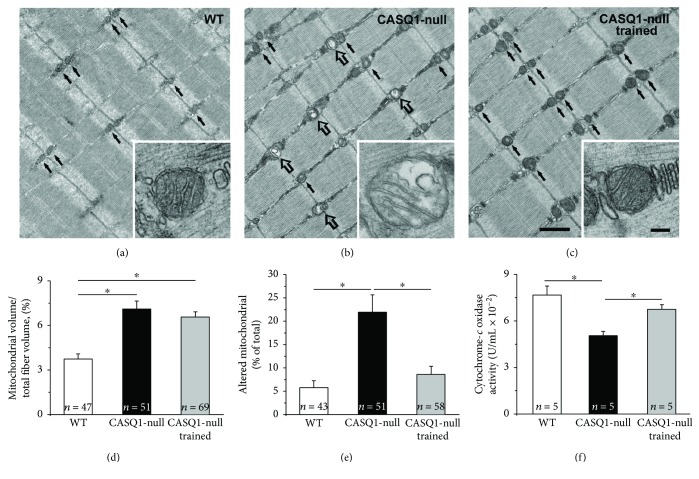
Structural and quantitative analysis of mitochondria. (a–c) Representative electron micrographs (longitudinal sections) of EDL fibers where small black arrows point to healthy mitochondria (see enlargements in (a) and (c)), while empty arrows point to damaged mitochondria (see enlargement in (b)). Scale bars in (a–c), 1 *μ*m; insets, 0.1 *μ*m. (d) Quantitative analysis of the relative fiber volume occupied by mitochondria. (e) Quantitative analysis of damaged mitochondria calculated as percentage of total. (f) Cytochrome *c* oxidase activity assayed in TA muscles. Data in (d) and (e) are shown as mean ± SEM; ^∗^*p* < 0.05, as evaluated by two-tailed unpaired Student's *t*-test for the 95% confidence intervals. Data in (f) are shown as mean ± SD; ^∗^*p* < 0.05, as evaluated by one-way ANOVA followed by Tukey's post hoc test. *n* = number of fibers analyzed (in panels (d) and (e)) and number of mice (in panel (f)).

In EDL fibers of WT mice, mitochondria are usually positioned at the I band in proximity of Z lines (Figures 3(a)–3(c) black arrows; see [60]). Healthy mitochondria usually exhibit an electron dense dark matrix (inset in Figure 3(a)), while when damaged, they would appear swollen and with a clear matrix [61]. A first qualitative assessment suggested that damaged mitochondria were more numerous in untrained CASQ1-null than in WT mice (Figure 3(b), empty arrows), but again rare in trained CASQ1-null mice (Figure 3(c)). The visual observations were supported by the quantitative analysis: (i) the relative volume occupied by mitochondria and (ii) the percentage of mitochondria presenting structural alterations were significantly higher in untrained CASQ1-null (6.6 ± 0.6% and 21.6 ± 3.8%) than in WT mice (3.9 ± 0.4% and 5.8 ± 1.4%) (Figures 3(d) and 3(e)). On the other hand, in trained CASQ1-null mice, while the relative volume occupied by mitochondria did not change significantly compared to untrained CASQ1-null mice (6.2 ± 0.6% versus 6.6 ± 0.6%), the percentage of damaged mitochondria was significantly reduced to a value close to that observed in WT muscles (8.6 ± 1.4% versus 5.8 ± 1.4%; see above) (Figures 3(d) and 3(e)). The structural improvement of mitochondrial structure would also suggest improved functional properties of mitochondria. Indeed, when we measured cytochrome *c* oxidase activity, we found that it was decreased in untrained CASQ1-null mice (0.05 ± 0.006 U/mL) compared to WT (0.076 ± 0.01 U/mL), but rescued to values closer to those of WT following training (0.067 ± 0.006 U/mL; Figure 3(f)). The data collected in the analysis of mitochondria could have implications for (i) the improved functional output (Table 1) and (ii) the higher capability of trained CASQ1-null mice to remove lactate (Figure 2).

### 3.3. Aerobic Training Protects Male CASQ1-Null Mice from Heat-Induced Sudden Death by Reducing Hyperthermia

**Figure 4:**
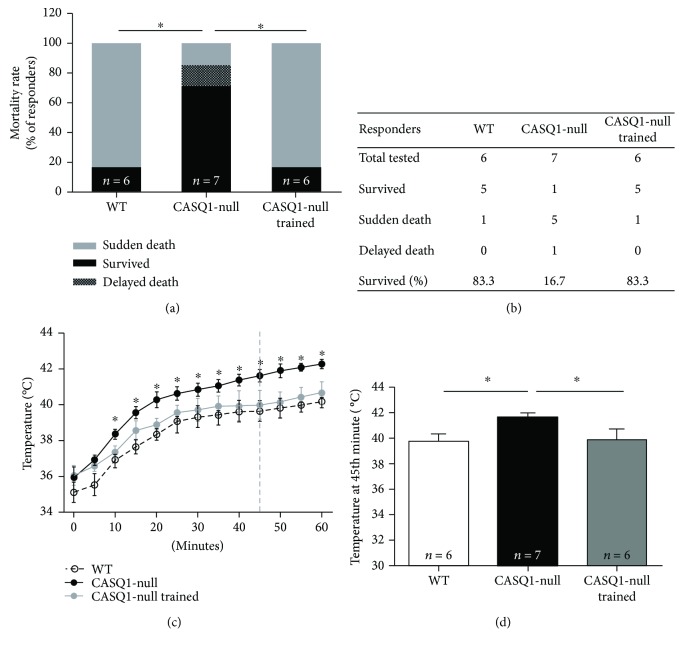
Mortality rate and measurements of core temperature in mice exposed to heat stress. (a) Incidence of sudden and delayed deaths (i.e., within 24 h after challenge) following exposure to the heat stress protocol (41°C for 1 h); ^∗^*p* < 0.05, as evaluated by two-tailed Fisher's exact test. (b) Table showing (i) the number of mice tested during the heat challenge and (ii) the experimental outcome. (c) Increase in absolute core temperature, recorded every 5 minutes during exposure to the heat stress protocol: dashed vertical line represents the mean time-to-onset of lethal crises in CASQ1-null mice; ^∗^*p* < 0.05, trained CASQ1-null versus both WT and trained CASQ1-null mice, as evaluated by two-way ANOVA followed by Bonferroni's post hoc test. (d) Average core temperature in mice at 45th minute of the heat stress protocol (dashed line in panel (c)). Data in (c) and (d) are given as mean ± SD; ^∗^*p* < 0.05, as evaluated by one-way ANOVA followed by Tukey's post hoc test. *n* = number of mice (in panel (d)).

Three days after the constant load test, all mice were subjected to a heat challenge in an environmental chamber (41°C for 1 h). Consistent with previous publications [21, 23], mortality rate during the heat stress protocol was significantly higher in CASQ1-null than in WT mice: 86% versus 18%, respectively (Figures 4(a) and 4(b)). Though, the mortality rate of CASQ1-null mice was reduced to only 16% after two months of aerobic training (Figures 4(a) and 4(b)). As a typical MH crisis is characterized by an abnormal rise in body temperature, namely, hyperthermia [1], we also monitored the core temperature throughout the entire duration of the heat challenge. Whereas core temperature raised in all animals (including WT) during the experiment, this increase was significantly greater in untrained CASQ1-null mice than in the other two groups of animals tested (Figure 4(c)). Specifically, the temperature calculated at 45th minute of the protocol, that is the average time-to-onset of hyperthermic crises in CASQ1-null mice [18, 23], was (i) on average 1.85°C higher in untrained CASQ1-null than in WT mice (Figure 4(d)) and (ii) on average 1.79°C lower in trained CASQ1-null than in untrained CASQ1-null mice, close to the temperature observed in WT animals (Figure 4(d)).

### 3.4. Aerobic Training Normalizes IVCT in CASQ1-Null EDL Muscles

**Figure 5:**
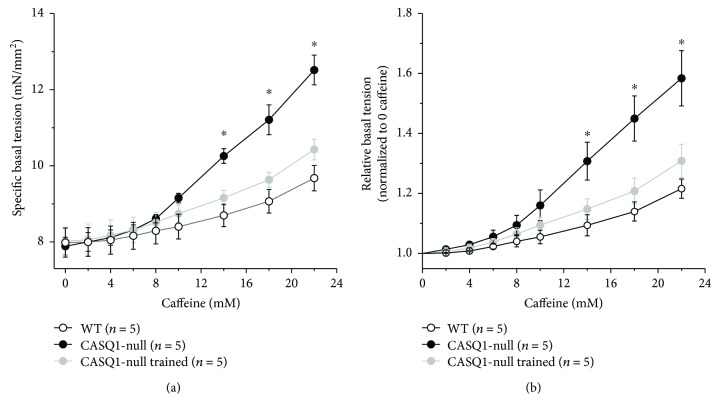
Caffeine dependence of basal tension in isolated EDL muscles during IVCT. (a) Specific basal tension of intact EDL muscles, recorded during exposure to increasing concentrations of caffeine. (b) Relative basal tension normalized to control conditions (no caffeine), during exposure to increasing concentrations of caffeine (same EDL muscles shown in panel (a)). Data are given as means ± SEM; ^∗^*p* < 0.05, as evaluated by ANOVA repeated measures, followed by Tukey's post hoc test for the pairwise comparisons. *n* = number of mice tested.

Intact EDL muscles were dissected from all mice and subjected to IVCT, the gold standard for the diagnosis of MH susceptibility in humans [62, 63]. Basal force of EDL muscles was measured during exposure to increasing concentrations of caffeine, a potent agonist of RyR1 that triggers release of Ca^2+^ from the SR. Force is displayed in Figures 5(a) and 5(b) as absolute and relative basal tension, respectively. While no differences in the specific basal tension were recorded among the three groups of animals in the absence of caffeine (7.9 ± 0.4 mN/mm^2^, 7.8 ± 0.2 mN/mm^2^, and 8.0 ± 0.4 mN/mm^2^ for WT untrained and trained CASQ1-null mice, resp.), when exposed to the IVCT protocol, EDL muscles from untrained CASQ1-null mice exhibited a greater caffeine sensitivity compared to those observed from WT (Figure 5). Specifically, EDL muscles from untrained CASQ1-null mice started to develop tension already at 10 mM and reached a final tension value of 12.5 ± 0.4 mN/mm^2^ at 22 mM of caffeine. Two months of aerobic training normalized the responsiveness to caffeine of CASQ1-null EDL muscles to values similar to that observed in WT: at 22 mM of caffeine, the specific basal tension reached by EDL muscles from WT and trained CASQ1-null mice was 9.7 ± 0.3 mN/mm^2^ and 10.4 ± 0.3 mN/mm^2^, respectively (Figure 5(a)). In Figure 5(b), force is displayed as relative basal tension normalized to control conditions (no caffeine) during the exposure to increasing concentrations of caffeine.

### 3.5. Calpain Activity Was Reduced in Muscles of Trained CASQ1-Null Mice

**Figure 6:**
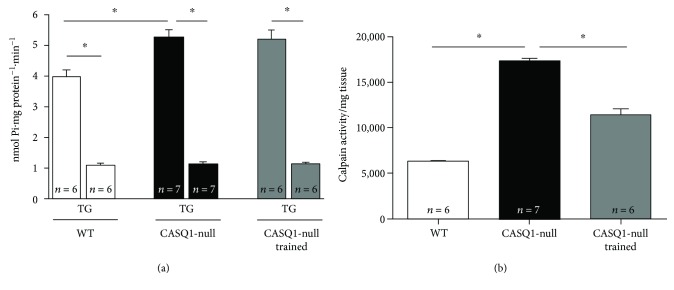
Measurements of SERCA and calpain activities. (a) SERCA activity assayed in microsomes isolated from gastrocnemius muscles in the absence and presence of 100 nM of thapsigargin (TG). (b) Calpain activity assayed in gastrocnemius muscles total homogenates. Data are given as mean ± SD; ^∗^*p* < 0.05, as evaluated by one-way ANOVA followed by Tukey's post hoc test. *n* = number of mice.

Cytosolic Ca^2+^ levels have been shown to be slightly elevated in CASQ1-null fibers [18, 21, 23] and may lead to an increased need of Ca^2+^ removal from the cytosol [37, 64]. For this reason, we measured SERCA activity in microsomal membranes of gastrocnemius muscles, estimated as production of P_i_ in the presence of ATP (Figure 6(a)). Our results showed that the rate of P_i_ generated in untrained CASQ1-null samples was significantly higher (32%) compared to that generated in WT. To demonstrate that this P_i_ generation was specifically dependent on the activity of SERCA, the same microsomes were treated with thapsigargin (TG, 100 nM), a noncompetitive inhibitor of SERCA [45]: after TG treatment, all samples exhibited a significant decay to ~1.0 nmol P_i_∙mg protein^−1^∙min^−1^, supporting the view of a different rate of SERCA activity in WT versus CASQ1-null fibers (Figure 6(a)). Though, SERCA activity was not reduced by aerobic training in CASQ1-null muscle. However, as increased levels of cytosolic Ca^2+^ may also result in an enhanced activation of proteolysis pathways [46], we also measured the total calpain activity, one of the most important nonlysosomal classes of proteases in skeletal muscle fibers, which is activated by chronic high Ca^2+^ levels [46, 65]. In this case, calpain activity, measured in total homogenates of gastrocnemius muscles, that was 2.7-fold higher in untrained CASQ1-null than WT specimens, was significantly lowered in trained CASQ1-null samples (Figure 6(b)).

### 3.6. Oxidative Stress Was Reduced by Aerobic Training in Muscles of CASQ1-Null Mice

**Figure 7:**
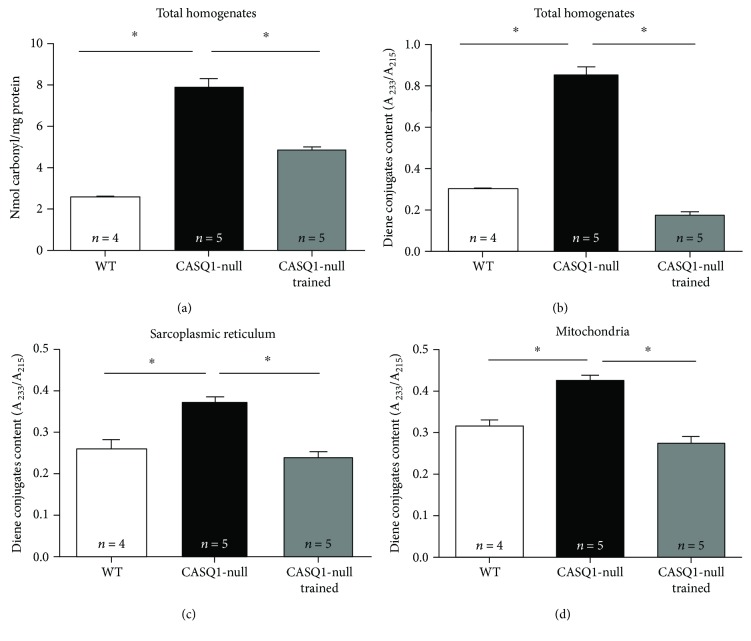
Oxidative modifications in proteins and membranes. Protein oxidation levels assayed by carbonyl protein content (a) and lipid peroxidation levels assayed by diene conjugate levels (b) in total homogenates from TA muscles. (c, d) Oxidative modifications assayed by diene conjugate levels in SR microsomes and in mitochondria isolated from gastrocnemius muscles. Data are given as mean ± SD; ^∗^*p* < 0.05, as evaluated by one-way ANOVA followed by Tukey's post hoc test. *n* = number of mice.

Oxidative stress has been shown to be elevated in CASQ1-null muscles [23]. To determine if aerobic exercise improved the capabilities of skeletal muscle to reduce oxidative damage, we evaluated (i) levels of carbonyl proteins (carbonyl protein content is an important biomarker of oxidative modification of proteins [53, 54]) and (ii) diene conjugates in total homogenates of TA muscle and in SR-and-mitochondria membranes of gastrocnemius muscles (diene conjugates are a by-product of lipid peroxidation chain, which is an indicator of structural oxidative modification to membranes [55]). The results obtained from these experiments revealed that (a) total carbonyl protein content was abnormally elevated in total muscle homogenates of untrained CASQ1-null compared to that of WT mice, while lowered of about 39% in muscles of trained CASQ1-null mice (Figure 7(a)); (b) levels of diene conjugates were significantly increased either in total homogenates or in isolated mitochondria and SR membranes obtained from untrained CASQ1-null compared to WT muscles but reduced of about 40% in all three preparations obtained from trained CASQ1-null muscles (Figures 7(b)–7(d)).

## 4. Discussion

In the last ten years, compelling evidence has been collected in animal models to demonstrate that EHS shares common molecular mechanisms with classic MH susceptibility, the reaction caused by exposure to halogenated anesthetics. Indeed, we and others have shown that knock-in mice carrying mutations in *RYR1* linked to MH in humans (RYR1^Y522S/WT^ mice) and CASQ1-null mice trigger lethal crises when exposed to anesthetics, heat, and also physical exertion [16–18, 21]. The molecular events leading to rhabdomyolysis of skeletal fibers during hyperthermic crises in both mouse models involve Ca^2+^ leak from RyR1 and excessive production of ROS/RNS, which then feeds a feedforward cycle that promotes additional SR Ca^2+^ release [24]. We demonstrated that this cycle can be interrupted by administration of antioxidants (*N*-acetylcysteine and trolox) [23, 24]. Here, we tested if aerobic training (Figure 1) can break the vicious cycle triggered by overproduction of ROS/RNS by boosting endogenous antioxidant defenses and, hence, prevent heatstrokes in CASQ1-null mice exposed to high environmental temperatures.

### 4.1. Main Findings of the Study

Our results show that aerobic training (a) protects mice from hyperthermic episodes, lowering both the increase in core temperature *in vivo* and the responsiveness of intact EDL muscles during IVCT (Figures 4 and 5), and (b) reduces oxidative stress, hence reducing mitochondrial damage (Figures 3, 6, and 7). Additionally, we also show that aerobic training had improved *in vivo* muscle performance, as shown by higher grip strength and maximal speed reached in the incremental test and by the reduced lactate accumulation in the bloodstream during the constant load test (Table 1 and Figure 2). These main points are discussed below in the same order.

#### 4.1.1. Protection from Lethal EHS Episodes

The training program in CASQ1-null mice resulted in a striking protection from heat-induced lethal episodes, with the survival rate raising from 17% in untrained to 83% in trained CASQ1-null mice (Figure 4). The protective effect of aerobic training is likely the direct result of (i) a reduced rise in core temperature (i.e., hyperthermia) in trained versus untrained CASQ1-null animals (Figure 4) and (ii) a normalized IVCT (Figure 5), which is an indirect, but quite reliable, measurement of Ca^2+^ handling, as muscle tension directly correlates with intracellular Ca^2+^ concentration. The fact that intracellular Ca^2+^ levels are slightly elevated in CASQ1-null mice has been demonstrated and discussed in depth in several previous publications [18, 21, 23, 61]. The elevated intracellular Ca^2+^ levels in untrained CASQ1-null muscles are here underlined both by IVCT (see above) and by increased SERCA and calpain activities (Figure 6). Indeed, both SERCA and calpain have been demonstrated to have their activities increased under conditions in which myoplasmic Ca^2+^ concentration is elevated [46, 47, 66]. Although aerobic training did not reduce the activity of SERCA, it did lower that of calpain, an effect that could contribute to several aspects presented in this work (see below).

#### 4.1.2. Protection from Oxidative Stress and Damage

Aerobic training resulted in a striking protection from heat-induced lethal episodes in CASQ1-null mice (Figure 4). Noticeable, a similar protection against EHS was also achieved treating CASQ1-null animals with antioxidants [23], or with estrogens [67], the primary female sex steroid hormones which have been shown to exhibit antioxidant properties [68–71]. Here we show that aerobic training, similarly to antioxidants and estrogens, reduces oxidative stress in CASQ1-null mice. Specifically, we demonstrated that protein carbonylation and diene conjugate formation, well-established markers of oxidative damage to proteins [53] and of lipid peroxidation [55], are greatly reduced in CASQ1-null mice subjected to aerobic training (Figure 7). This effect is possibly achieved, thanks to the increased endogenous antioxidant protection, as lipid peroxidation is strongly influenced by endogenous antioxidant defenses [55, 56]. The fact that aerobic training reduces oxidative stress is not surprising: the training protocol used in the present study (Figure 1) was indeed demonstrated to increase aerobic capacity and to counteract protein and lipid oxidative modifications in skeletal muscles of heart failure-bearing animals [34]. In addition, many investigators have reported that exercise training boosts antioxidant defenses [33, 34, 72–74] while decreasing lipid peroxidation [29] and oxidative modifications to proteins and RNA [55].

#### 4.1.3. Adaptations of CASQ1-Null Mice to Exercise

Muscle function and lactate production were evaluated in all three groups of mice (Table 1) and allowed to determine differences caused by adaptation of muscle to lack of CASQ1 (control CASQ1-null versus WT) and to training (control versus trained CASQ1-null). While grip strength test indicated that control CASQ1-null mice are weaker than age-matched WT (Table 1), a result in accordance with data previously published [23, 61], the maximal speed reached during the incremental test was greater in knockout animals. While surprising, this finding is possibly explained by the much greater content of mitochondria in muscle fibers from CASQ1-null mice (Figure 3 and [27]) and by the higher capability of knockout animals to remove lactate from their bloodstream (Figure 2). On the other hand, not surprising was the fact that aerobic training in CASQ1-null mice improved both the grip strength and the performance in the incremental test (Table 1). The improved capabilities of CASQ1-null following training may be the direct result of (i) the reduced blood lactate accumulation (Figure 2) and of (ii) the improved mitochondrial structure and function (Figure 3).

### 4.2. Altered Mitochondria Are a Potential Source of Excessive Oxidative Stress

Generation of free radicals inside the cells may arise from multiple sources, including mitochondria [75]. The enzyme cytochrome *c* oxidase is a transmembrane protein found in the inner mitochondrial membrane, crucial for ATP synthesis [52], a process that can produce superoxide anion (O_2_^•−^) [76]. Production of oxidative species may increase once mitochondria are damaged [76, 77]. Indeed, in untrained CASQ1-null muscle fibers, mitochondria are more frequently damaged than in WT while activity of cytochrome *c* is reduced and oxidative stress is elevated (Figures 3 and 7). In previous publications, we already showed elevated mitochondrial damage and increased levels of superoxide dismutase 1 and 2, respectively, the cytosolic and the mitochondrial isoforms [23, 67]. Present data also show that aerobic training in CASQ1-null mice reduces mitochondrial damage while increasing cytochrome *c* oxidase activity, likely as the consequence of reduced calpain activity (Figure 6) and oxidative stress (Figure 7). In turn, improved mitochondrial structure could also contribute to the improved muscle performance (Table 1).

### 4.3. Closing Remarks

In latest years, global warming has become reason of health concern [78, 79] because of (a) the increased frequency and severity of unusual heat waves (i.e., period of abnormally high temperatures and humidity) [78] and (b) a dramatic rise in the mortality rate due to heat-related illnesses during these heat waves [80, 81]. The impact of heat waves is especially severe in urban areas and well documented in literature (more than 1000 scientific reports). Surprisingly, these reports indicate that 95% of human deaths due to natural hazards are caused by hot and humid weather [82, 83]. The 2003 heat wave in France, one of the better described in literature, was accompanied by an excess mortality of exceptional magnitude: 14,947 excess deaths for the period of August 4–18. Interestingly, mortality rate of the population returned to its normal level starting on August 19 [84]. Also striking was the rate of mortality in the 1995 heat wave in Chicago [81]. A recent report indicates that the effects of high temperatures over consecutive days on human health are quite similar to those caused by single unusually hot days [85]. Even if several factors may contribute to the death caused by high environmental temperatures (age, preexisting disease, urban residence, isolation, poverty, and air pollution), the most common cause of death attributable to heat is dehydration, heat cramps and exhaustion, and hyperthermia, that is, in one word EHS [85]. EHS is life-threatening mainly because skeletal muscle represents a high percentage of the human body mass, and even rhabdomyolysis of a small percentage of fibers will cause a significant modification of the blood parameters, which in turn may challenge heart and kidney functions [1]. Understanding the molecular mechanisms underlying EHS, and dissect which molecular pathways must be interrupted to prevent/revert crises, is crucial. Also urgent is the development of (a) drugs to be used in emergency situations and (b) life habits that may prevent the triggering of EHS in hot weather. Here we have shown that in CASQ1-null mice, which are susceptible to both exertional and environmental heatstroke [18, 21, 23], aerobic training significantly reduced the mortality rate during exposure to heat by lowering oxidative stress. This knowledge may help in the future to develop guidelines for those populations on earth that are frequently exposed to high environmental temperatures, hence to the risk of EHS.

## Figures and Tables

**Table 1:** Body weight and functional output of mice before and after training.

	Pretraining (2 months of age)		Posttraining (4 months of age)		
	WT (*n* = 7)	CASQ1-null (*n* = 21)	Untrained		Trained
			WT (*n* = 7)	CASQ1-null (*n* = 14)	CASQ1-null (*n* = 7)
Body weight (g)	24.6 ± 2.9	23.6 ± 2.5	29.9 ± 2.8	27.0 ± 2.9	25.7 ± 3.0
Grip strength normalized (peak force g/g weight)	9.7 ± 1.5	3.9 ± 1.6^∗^	7.9 ± 1.7	3.0 ± 0.8^†^	4.8 ± 1.1^#^
Max. speed in the incremental test (m/min)	24.8 ± 3.1	27.8 ± 3.0^∗^	24.0 ± 2.1	28.1 ± 2.7^†^	32.5 ± 3.9^#^

Data are shown as mean ± SD. ^∗^*p* < 0.05 compared to age-matched WT, as evaluated by two-tailed unpaired Student's *t*-test for the 95% confidence intervals; ^†^*p* < 0.05 when compared to WT at 4 months, and ^#^*p* < 0.05 compared to untrained CASQ1-null, as evaluated by one-way ANOVA followed by Tukey's post hoc test. All groups were tested for normal distribution by Kolmogorov-Smirnov test and outlier detection by Grubb's test.

## Abbreviations

CASQ1: Calsequestrin type-1
CASQ1-null: CASQ1 knockout mice
CK: Creatine kinase
EDL: Extensor digitorum longus
EHS: Exertional/environmental heatstroke
EM: Electron microscopy
IVCT: *In vitro* contracture test
MH: Malignant hyperthermia
RyR1: Ryanodine receptor type-1
SERCA: Sarcoplasmic/endoplasmic reticulum Ca^2+^ ATPase
SR: Sarcoplasmic reticulum
TA: Tibialis anterior
WT: Wild type.
